# Supplementary material for: Diversity of Short Linear Interaction Motifs in SARS-CoV-2 Nucleocapsid Protein
Source: bioRxiv. 2023 Aug 1:2023.08.01.551467. Preprint. [Version 1] doi: 10.1101/2023.08.01.551467 (PMC10542142; doi:10.1101/2023.08.01.551467)
Supplement: Supplement 1 [file media-1.pdf]

SUPPLEMENARY INFORMATION

Diversity of Short Linear Interaction Motifs in SARS-CoV-2 Nucleocapsid Protein

Peter Schuck* and Huaying Zhao

Laboratory of Dynamics of Macromolecular Assembly, National Institute of Biomedical Imaging and Bioengineering, National Institutes of Health, Bethesda, MD 20892, USA

*Correspondence to schuckp@mail.nih.gov

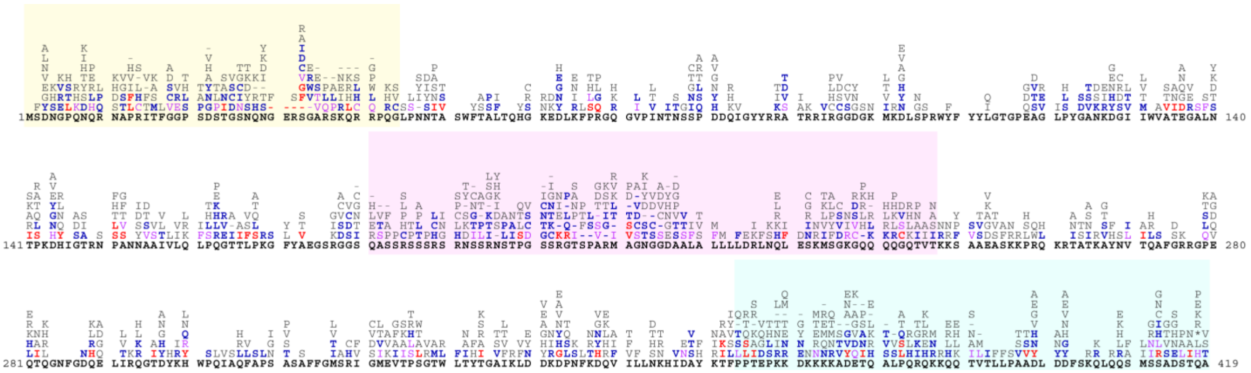

Figure S1. Mutational landscape of SARS-CoV-2 Omicron N-protein

As in **Figure 1**, shown in bold black letters is the ancestral amino acid sequence (Wuhan-Hu-1), and on top are mutations found in 5.05 million sequences from the GISAID repository, downloaded from Nextstrain in January 2023, and classified as one of the Omicron clades. The mutations are ordered and colored by number of genomes found to contain that specific mutation: 10 to 100 (gray), 100 to 500 (blue), 500 to 1,000 (purple), and > 1,000 (red). The intrinsically disordered regions are highlighted: N-arm in yellow, the linker in magenta, and C-arm in cyan.
